# Clinical guide SEOM on venous thromboembolism in cancer patients

**DOI:** 10.1007/s12094-014-1238-y

**Published:** 2014-11-01

**Authors:** A. J. Muñoz Martín, C. Font Puig, L. M. Navarro Martín, P. Borrega García, M. Martín Jiménez

**Affiliations:** 1 Medical Oncology Service, Gregorio Marañón University General Hospital, Madrid, Spain; 2 Servicio de Oncología Médica-Hospital de Día, Hospital Clinic i Provincial, Barcelona, Spain; 3 Medical Oncology Service, University Hospital of Salamanca, Salamanca, Spain; 4 Medical Oncology Service, Cáceres Hospital Complex, Cáceres, Spain

**Keywords:** Cancer, Thrombosis, Guidelines, Anticoagulation

## Abstract

Venous thromboembolism (VTE) is a common event in cancer patients and one of the major causes of cancer-associated mortality and a leading cause of morbidity. In recent years, the incidence rates of VTE have notably increased; however, VTE is still commonly underestimated by oncologists. VTE is considered an adverse prognostic factor in cancer patients in all settings. In 2011 the Spanish Society of Medical Oncology (SEOM) first published a clinical guideline of prophylaxis and treatment of VTE in cancer patients. In an effort to incorporate evidence obtained since the original publication, SEOM presents an update of the guideline for thrombosis and cancer in order to improve the prevention and management of VTE.

## Introduction

Venous thromboembolism (VTE) is a significant cause of morbidity and death in patients with cancer. Although deep vein thrombosis (DVT) of limbs and pulmonary embolism (PE) are the most commonly encountered venous thrombotic complications, other vascular territories, such as the splanchnic veins and central nervous system can be involved. The risk of VTE is estimated to be fourfold higher in cancer patients compared with non-cancer patients. The true incidence of VTE in cancer patients remains uncertain, with reported incidence rates ranging from 0.8 % to over 30 % in some populations, and depends on several factors (tumor, host and treatment-related factors) [1]. VTE is often asymptomatic or minimally symptomatic, therefore the incidence is likely to be much higher. A steady increase in the incidence of cancer-associated thrombosis (CAT) has been observed in the past two decades due to multiple factors: increasing age of general population and cancer prevalence, improved imaging techniques with enhanced detection of incidental thrombosis and greater thrombogenicity of current multiagent chemotherapy regimens. Cancer patients with VTE have an increased incidence of VTE recurrence and anticoagulant-related bleeding complications compared with patients without cancer. VTE has been found to be an adverse prognosis factor in all stages of cancer [2]. In the last decade it was postulated that the aggressiveness of a malignant tumor is clearly correlated to the incidence of VTE.

Here, we review the prophylaxis and treatment of VTE in cancer patients using the GRADE system for all the recommendations [3, 4].

## Risk factors

The risk of VTE varies notably between cancer patients, even in the same patient over the course of the disease. Risk factors for CAT can be divided into three categories: patient-, treatment- and cancer- related factors (Table 1).

**Table 1:** Risk factors for VTE

Cancer-related
Site
Very high risk: pancreas, brain, stomach
High risk: lung, kidney, colon, uterus, bladder, testicular tumor
Low risk: prostate, breast
Stage/metastatic disease
Higher for metastatic disease over locally advanced or local disease
Histology
Higher for adenocarcinoma over squamous cell carcinoma
Tumor grade
Higher for high-grade tumors (grade 3–4) compared to low grade (grade 1–2)
Initial period after diagnosis (3–6 months)
Active disease
Vascular compression due to tumoral mass or lymphadenopathy
Treatment-related
Chemotherapy
Cisplatin
Surgery
Hospitalization
Hormonal treatment
Indwelling catheters
Glucocorticoids
Transfusions
Erythrocyte and platelet transfusions
Erythropoietic stimulating agents
Antiangiogenic agents
Thalidomide and lenalidomide
Patient-related
Older age
>65 years
Obesity
>35 BMI
African-American
Female
Prior VTE history
Chronic venous insufficiency
Comorbidities/medical problems (infection, pulmonary or renal disease, arterial thromboembolism, others)
Pregnancy
Tobacco
Poor performance status
Low level of activity/physical exercise
Major trauma and immobilization
Inherited thrombophilia (Factor V Leiden)

*BMI* body mass index

## Prophylaxis

### Prophylaxis of VTE in hospitalized medical cancer patients

Hospitalization is one of the main VTE risk factors with surgery and trauma. Medical hospitalized patients are at significant risk of developing VTE and thromboprophylaxis has been shown to be effective in three large randomized phase III trials [5–7]. All three studies reported a significant reduction in VTE following treatment with low-molecular-weight heparins (LMWH) or fondaparinux, compared with placebo. However, there is a lack of evidence regarding hospitalized cancer patients, because no specific trials have ever been conducted in the cancer population. The only evidence available is the subgroup analysis of the aforementioned trials (Table 2). LMWH trials showed similar VTE reductions; however, an increase incidence of VTE paradoxically was observed with fondaparinux. Major bleeding rates were not reported in the three placebo-controlled trials. A recent meta-analysis [8] of the cancer population in these three studies showed that cancer patients did not gain a significant reduction in the incidence of VTE when pharmacological anticoagulation was used. Different explanations have been suggested for this finding as lack of statistical power, small number of patients included in the analysis (307 patients), lack of stratification according to VTE risk or cancer status, heterogeneity between studies, lack of efficacy using standard doses of drug prophylaxis (higher doses in this high-risk population) or low-risk patients included in these trials. Finally, it has been suggested that fondaparinux is less efficacious than LMWH.

**Table 2:** Clinical trials assessing prophylaxis of VTE in hospitalized medical patients

Clinical trial	Number of patients	Cancer patients (%)	Study drugs	VTE events	Relative risk reduction	Major bleeding	NTT	Cancer subgroup VTE events
ARTEMIS	849	15.4	Fondaparinux sc (2.5 mg/24 h) vs. placebo	5.6 vs. 10.5 % *p* = 0.029	0.47	0.2 vs. 0.2 % *p* = NS	20	17.0 vs. 3.9 % RR 4.3 NNH 8
MEDENOX	866	12.4	Enoxaparin sc (40 mg/24 h) vs. placebo	5.5 vs. 14.9 % *p* < 0.001	0.37	1.7 vs. 1.1 % *p* = NS	11	9.7 vs. 19.5 % RR 0.50 (95 % CI 0.14–1.72) NNT 10
PREVENT	3,706	5.1	Dalteparin sc (5,000 UI/24 h) vs. placebo	2.8 vs. 5.0 % *p* = 0.0015	0.55	0.5 vs. 0.2 % *p* = NS	45	3.1 vs. 8.3 % RR 0.37 NNT 18

*sc* subcutaneously, *VTE* venous thromboembolism, *NS* not significant, *NTT* number of patients needed to treat to avoid one event, *NNH* number needed to harm, *RR* relative risk, *CI* confidence interval

Some observational studies suggest that VTE risk in cancer patients extends beyond their hospital stay; however, there is no evidence to recommend extended thromboprophylaxis after discharge. No specific trials have been performed with the new oral anticoagulants (NOACS) in this setting in cancer patients. To date there is a lack of validated risk assessment tools for estimating the overall risk of VTE and bleeding in hospitalized cancer patients. Identifying patients who could benefit most from pharmacologic prophylaxis and performing targeted thromboprophylaxis are important issues for specialists caring for cancer patients.

#### *Recommendation*

Despite the paucity of data, prophylactic anticoagulation should be considered for hospitalized cancer patients with acute medical illness in the absence of contraindications. The preferred agents are LMWH (level of evidence: grade 1B). There is no evidence to recommend NOACS or extended prophylaxis after hospital discharge.

### Prophylaxis of VTE in surgical cancer patients

VTE is a common complication in cancer patients undergoing surgery. Cancer surgery doubles the risk of DVT and the risk of fatal postoperative PE is four times higher compared to similar procedures in the non-cancer population. In addition, cancer surgery is associated with an increased risk of bleeding. Several randomized studies and meta-analyses have demonstrated the benefit of pharmacologic prophylaxis in this setting with LMWH and UFH over no prophylaxis or placebo. Pharmacologic prophylaxis is ideally started before surgery or as soon as possible in the postoperative period. Multiple trials in unselected populations including cancer and non-cancer patients suggest LMWH and UFH are equally effective. Due to similar efficacy and unfavorable schedule (three times a day vs. once a day), LMWH is preferable to UFH in surgical cancer patients. There is a lack of data of the superiority of one type of LMWH over another. Classically prophylaxis is continued for at least 7–10 days. It must be noted 40 % of the VTE events may occur later than 21 days from surgical intervention [9]. Prolonged prophylaxis for up to 4 weeks must be considered in patients undergoing major abdominal or pelvic surgery for cancer with additional risk factors (Table 1) [10]. Mechanical methods of thromboprophylaxis in monotherapy modestly reduce the frequency of VTE in cancer patients. Few data are available from prospective, randomized, controlled trials on their efficacy in cancer patients. Pharmacological thromboprophylaxis is superior to mechanical thromboprophylaxis in preventing VTE [11]. So mechanical methods are commonly used as an adjunct to pharmacological thromboprophylaxis. A very important advantage of mechanical methods is they are not associated to an increased risk of bleeding. The optimal prophylaxis for laparoscopic surgery or minimally invasive procedures has not been established and the real incidence of VTE is not well known. The need for pneumoperitoneum and reverse Trendelenburg position during laparoscopic procedure may increase the incidence of VTE in these patients. Observational studies reported laparoscopic surgery was associated with a lower incidence of VTE compared to open surgery. A recent Italian study published in 2013 showed the superiority of extended prophylaxis for 4 weeks over short prophylaxis (1 week) in patients who underwent laparoscopic surgery for colorectal cancer.

#### *Recommendation*

In the absence of contraindications, all patients undergoing major surgical intervention should receive pharmacologic thromboprophylaxis (level of evidence: grade 1A). The preferred agents are LMWH and prophylaxis should be started before surgery or as soon as possible in the postoperative period. Mechanical methods may be added to pharmacologic prophylaxis in high-risk patients but should not be used as monotherapy, unless pharmacologic prophylaxis is contraindicated (level of evidence: grade 2C). Patients should receive at least 7–10 days of prophylaxis and patients undergoing major abdominal or pelvic cancer surgery with high-risk features should be considered for extended thromboprophylaxis for 4 weeks (level of evidence: grade 1A).

We suggest the same recommendations for laparoscopic surgery, risk factors and the duration and type of the procedure must be assessed (level of evidence: grade 2C).

### Prophylaxis of VTE in ambulatory cancer patients during chemotherapy

Chemotherapy has been identified as an independent risk factor for VTE. The rates of thrombosis in ambulatory patients receiving chemotherapy vary widely and depend on multiple factors (see Table 1). Several randomized trials evaluating ambulatory prophylaxis with LMWH and low-dose warfarin in ambulatory cancer patients have been published with inconsistent results largely due to the heterogeneity of the populations studied and the treatments used (duration, drugs, dose, etc.). Since 2009 four randomized trials [12–15] and three meta-analyses [16–18] have been published and have changed the landscape of thromboprophylaxis in this setting (Table 3). All studies randomized patients with cancer receiving chemotherapy to venous thromboprophylaxis or placebo. These studies consistently demonstrate the benefit of thromboprophylaxis in different types of malignancies with a significant reduced risk of VTE with an acceptable safety profile. However, the overall rates of VTE among patients assigned to placebo were very low, ranging 3–4 % in the multi-tumor trials which is lower than observed in the real world [12, 13]. The lower VTE incidence rates have been suggested by the inclusion of selected low-risk patients. Therefore, despite a consistent and robust reduction in the risk of VTE with a hazard ratio under 0.4 in these studies, the difference in absolute risk is small. The rate of major and minor bleeding was similar between the two arms. Even though there was a decrease in the VTE incidence no difference in overall survival was observed. The effect of this type of thromboprophylaxis on quality of life has not been assessed. According to the evidence from these trials and Cochrane meta-analysis the number of patients needed to treat to avoid one VTE event (NNT) would range between 46 and 60. In consequence, all these issues and the cost of LMWH have limited the expansion of thromboprophylaxis in the ambulatory setting. A pooled analysis [18] that included more than 6,000 patients showed a significant reduction in symptomatic VTE (relative risk 0.57, 95 % CI 0.40–0.81) and a non-significant effect on major or minor bleeding (Table 3). This analysis also suggested a small survival benefit for prophylaxis with LMWH (relative risk 0.94, 95 % CI 0.88–1.00) not observed in the previous studies [18].

**Table 3:** Prophylaxis of VTE in ambulatory cancer patients during chemotherapy: recent clinical trials and meta-analysis

Study	Number of patients	Type of tumor	Risk of thrombosis	LMWH	Dose
PROTECHT Lancet Onc’09	1,150	Lung, pancreas, stomach, colorectal, breast, ovarian, head and neck cancer	High (pancreas, stomach) Low (breast, head and neck)	Nadroparin	3,800 UI/24 h
FRAGEM UK EJC’11	123	Pancreas	High	Dalteparin	200 UI/kg/24 h × 4 weeks followed 150 UI/kg/24 h × 8 weeks
CONKO 004 ASCO’10	312	Pancreas	High	Enoxaparin	1 mg/kg/24 h × 3 m, followed 40 mg/24 h × 3 m
SAVE ONCO NEJM’12	3,212	Lung, colorectal, stomach, pancreas, kidney and ovarian cancer	Moderate–high	Semuloparin	20 mg/24 h
Meta-analysis Cochrane 2012	3,538	Multiple neoplasms	Not defined	–	–
Akl pooled analysis NEJM’12	≈6.000	Multiple neoplasms	Not defined	–	–

Duration	VTE (%) CT + LMWH vs. CT	Major bleeding CT + LMWH vs. QT	Minor bleeding CT + LMWH vs. CT	NNT
4 months	2.0 vs. 3.9 % *(VTE + ATE) *p* = 0.02	0.7 vs. 0 % *p* = 0.18	7.4 vs. 7.9 %	53
12 weeks	3.4 vs. 23.0 % RR 0.145, *p* = 0.002	3.4 vs. 3.2 %	9.0 vs. 3.0 %	–
6 months	5.1 vs. 15.6 % *p* < 0.05	No difference *p* = NS	NR	12 (sVTE)
Until a change of CT regimen	1.2 % vs. 3.4 % HR 0.36, *p* < 0.001	1.2 vs. 1.2 %	1.6 vs. 0.9 %	46
–	Heparin vs. no prophylaxis			60 (sVTE)
	0.55 (0.34–0.88)	1.57 (0.69–3.60)	–	
–	Heparin vs. no prophylaxis			–
	0.57 (0.40–0.81)	1.06 (0.71–1.57)	1.18 (0.89–1.55)	

*m* months, *mg* milligram, *CT* chemotherapy, *NNT* number of patients needed to treat to avoid one event, *sVTE* symptomatic venous thromboembolism, *NS* not significant, *HR* hazard ratio

* Venous thromboembolism incidence plus arterial thormboembolism incidence

The risk of VTE in patients diagnosed with multiple myeloma receiving treatment with thalidomide or lenalidomide plus chemotherapy or dexamethasone ranges from 12 to 28 %. When any pharmacologic thromboprophylaxis strategy is scheduled, this figure drops below 10 %. There is a lack of randomized studies in patients with multiple myeloma treated with thalidomide or lenalidomide in combination with chemotherapy or dexamethasone comparing VTE prophylaxis vs. observation. Two randomized controlled trials that compared different strategies of prophylaxis have been published [19, 20]. They showed aspirin and LMWH are acceptable thromboprophylaxis options and both suggested slightly greater efficacy of LMWH compared with aspirin.

It is important to identify patients at higher risk for whom prophylaxis may be beneficial. A validated risk assessment model (RAM) for identifying patients at high risk for VTE receiving chemotherapy has been validated by Khorana et al. [21]. Five predictive variables were identified and three different risk categories were defined (Table 4). This RAM is also highly predictive of mortality and progression-free survival. Recently, this model has been expanded with the addition of D-dimer and P-selectin. The addition of these biomarkers improves the risk prediction of VTE considerably; nevertheless, this expanded model has little impact since P-selectin is not available in clinical practice and has not been validated. In the near future new biomarkers may enhance the VTE prediction in this setting.

**Table 4:** Khorana’s risk assessment model (RAM)

Patient characteristics		Risk score points
Site of cancer		
Very high risk (stomach, pancreas)		2
High risk (lung, lymphoma, gynecologic, genitourinary excluding prostate)		1
Pre-chemotherapy platelet count ≥350,000/mm^3^		1
Hemoglobin level less than <10 g/dl or use of red cell growth factors		1
Pre-chemotherapy leukocyte count >11,000/mm^3^		1
BMI 35 ≥ 35 kg/m^2^		1

Risk score (points)	Risk category	Rates of sVTE according to scores (%)
0	Low	0.3*–*0.8
1–2	Intermediate	1.8–2.0
≥3	High	6.7–7.1

*BMI* body mass index, *sVTE* symptomatic VTE

#### *Recommendation*

Routine thromboprophylaxis is not recommended in ambulatory cancer patients receiving chemotherapy (level of evidence: grade 1B). LMWH may be considered in high-risk ambulatory cancer patients, as advanced pancreatic cancer or patients with a Khorana score ≥3, with low bleeding risk. Ambulatory patients who are receiving chemotherapy and prophylaxis with LMWH against VTE should be closely monitored.

It is recommended to assess the risk of VTE in all patients starting chemotherapy. Khorana’s score is the only validated RAM in cancer patients receiving chemotherapy and is the recommended tool to assess VTE risk. Risk assessments should be performed periodically throughout the patient’s chemotherapy (level of evidence: grade 2C).

Pharmacologic thromboprophylaxis is recommended in patients diagnosed with multiple myeloma receiving treatment with thalidomide or lenalidomide plus chemotherapy or dexamethasone. LMWH is recommended for high-risk patients and aspirin for low-risk patients (level of evidence: grade 2B).

### Prophylaxis of VTE in cancer patients with central venous catheters

Central venous catheters (CVC) are associated with upper extremity DVT and PE and are considered independent risk factors for VTE. Controversy remains regarding the true incidence of the disease, as many CVC-associated VTEs remain subclinical. The latest data suggest that while CVC-associated symptomatic VTE incidence only amounts to 2–5 %, asymptomatic VTE may be as high as 27–66 %, depending on the screening method used. In recent years a reduction of the CVC-associated VTE rate has been described. This finding is likely to be due to the introduction of less thrombogenic materials in the manufacturing of CVC. The most recent and largest studies and a meta-analysis of randomized trials did not show clinically meaningful degrees of protection against catheter-induced upper extremity venous thrombosis using either low-dose warfarin or LMWH in patients with cancer. Several studies including one meta-analysis [22] have suggested that CVC should be placed on the right side, in the jugular vein and the catheter tip should be positioned at the right atrium/superior vena cava junction.

#### Recommendation

Routine prophylaxis of VTE in cancer patients with CVC is not recommended. To reduce the incidence of VTE, CVC should be placed on the right side, in the jugular vein and the catheter tip should be positioned at the right atrium/superior vena cava junction (level of evidence: grade 1A).

## Treatment

The goals of anticoagulant therapy in cancer patients with CAT are to improve symptoms, reduce risk of recurrent VTE and decrease the risk of post-thrombotic syndrome (PTS). Anticoagulation is the cornerstone of treatment. Cancer patients present a higher risk of recurrent VTE and anticoagulant treatment-related bleeding compared to those without malignancy during anticoagulation therapy [23].

### Initial treatment of VTE in cancer patients (5–10 days)

There are no randomized trials to specifically assess the initial treatment of VTE in cancer patients. Available clinical evidence comes from subgroup analysis and a meta-analysis of 16 randomized studies developed in the general population [24]. These studies neither found no significant differences in the VTE recurrence rate between LMWH and UFH nor the incidence of bleeding. However, a significant reduction in mortality at 3 months in favor of LMWH compared with UFH (RR 0.71 CI 95 % 0.52–0.98) was shown. Initial treatment (5–10 days) of CAT with LMWH at a body weight-adjusted dose has become the drug of choice. LMWH administered subcutaneously is at least as safe and effective as UFH administered intravenously and LMWH usually requires no laboratory monitoring. Moreover, LMWH is associated with a reduced risk of developing heparin-induced thrombocytopenia (HIT) and osteoporosis compared to UFH. Alternative agents are UFH and fondaparinux according to the patient’s characteristics and clinical situation. Fondaparinux is similar to LMWH in terms of administration route, efficacy, costs and convenience, but available data from randomized trials about this drug in cancer patients are scarce, so its use must be restricted to avoid HIT and previous allergic reactions to heparins.

Due to the lack of data in cancer populations, the indications and contraindications for the initial use of thrombolytic therapy are essentially the same as for non-cancer patients. The indications and potential benefits must be carefully weighed against the risk of adverse effects for each patient, due to the higher risk of major and fatal bleeding. Generally, it is only advisable in the management of patients with life- or limb-threatening thrombotic events. Multiple thrombolytic agents and regimens have been used, but any superiority of a particular agent or regimen remains to be definitively established.

#### Recommendation

LMWH at a body weight-adjusted dose is the drug of choice for the initial treatment of CAT (level of evidence: grade 1A). Thrombolytic therapy is only recommended in patients with life- or limb-threatening thrombotic events (level of evidence: grade 2C).

### Treatment of central venous catheter-associated thrombosis (CVCAT)

The cornerstone of the treatment of CVCAT is anticoagulant therapy and anticoagulation without CVC removal is the preferred approach in this setting. The type, duration and intensity of anticoagulant therapy of upper extremity deep vein thrombosis (UEDVT) should be the same as in the strategy employed for DVT of the legs. It is recommended to remove the CVC if [25, 26]: the catheter is not functional or the catheter is no longer needed, failure of anticoagulation therapy is observed, anticoagulation is contraindicated or in catheter infection. Anticoagulation should continue for as long as the catheter is in place and for at least 3 months after catheter removal. For massive CVC-associated UEDVT and PE, thrombolytic therapy must be considered.

#### Recommendation

The treatment strategy of CVCAT should be the same as recommended in the treatment of DVT of the lower limbs (level of evidence: grade 2C).

### Treatment of acute PE

The clinical spectrum of PE ranges from an acute life-threatening event to a silent finding in scheduled CT scans. The strongest predictor for short-term PE-related mortality is hemodynamic status. In patients with hemodynamic instability the short-term mortality rate ranges from 15 to 60 % of cases and acute thrombolytic therapy should be considered in this setting after weighting the PE severity and the risk of bleeding. The use of the catheter or surgical embolectomy may also be considered in patients with massive PE with contraindications to thrombolytic therapy or in those that remain unstable after thrombolysis. However, the vast majority of patients with PE are hemodynamically stable at diagnosis. Anticoagulation with LMWH is currently the treatment of choice in patients with normotensive PE even in those with right ventricular dysfunction according to data from recent trials which have failed to demonstrate that the benefits of systemic thrombolysis outweigh the risk of bleeding in this setting. Several risk-stratification tools that include clinical decision rules, cardiac biomarkers or imaging tests have been developed to classify patients with normotensive PE. Of note, since cancer has been found to be an independent predictor of death in series of patients with acute symptomatic PE, general PE prognostic scales include cancer as a predictive variable limiting its use in the cancer population. This fact has led to the development of specific prognostic scales for acute symptomatic cancer-related PE including the POMPE-C tool [27] and a score derived from the Riete registry [28].

#### Recommendation

LMWH is the treatment of choice in patients with PE (level of evidence: grade 1A).

### Treatment of incidental PE

It is known that subclinical or asymptomatic PE may be present in up to 50 % of patients with acute DVT. Incidental or unsuspected VTE found during the evaluation of scheduled CT scan evaluations in cancer patients is common, accounting for 2–5 % of all CT scans. Incidental PE currently represents about 50 % of PE burden in cancer patients. Moreover, the incidence of incidental VTE is likely to increase further with the improvements of imaging techniques. Limited data from retrospective and observational cohorts of patients suggest that patients with incidental VTE would have similar outcomes with regard to overall survival and the risk of bleeding and VTE recurrence compared to patients with acute symptomatic events [29]. Unfortunately, there is a lack of evidence from prospective interventional studies to assess the best management of incidental VTE and whether anticoagulation is indicated or beneficial in patients with incidental VTE remains controversial. Expert recommendations suggested treating incidental VTE with standard full-dose anticoagulation therapy similarly to symptomatic events. However, in cases where the diagnosis of VTE is doubtful or questionable such as in cases of isolated subsegmental PE, additional confirming tests such as compression venous ultrasonography or CT scan pulmonary angiography should be performed.

#### Recommendation

Anticoagulation therapy is considered the standard treatment of incidental PE (level of evidence: grade 1C).

### Long-term treatment of CAT

The preferred agent for long-term management and secondary VTE prophylaxis (from first 5–10 days up to 3–6 months after the VTE diagnosis) in cancer patients is LMWH. This recommendation is based on several randomized controlled trials [30–33] and a meta-analysis of 7 studies [34] which have provided consistent evidence on improved efficacy of LMWH (dalteparin, enoxaparin and tinzaparin) compared to vitamin K (VKA) antagonists in this setting (Table 5). Overall, a relative risk reduction of 53 % in the prevention of recurrent VTE has been observed with the use of LMWH compared to VKA with similar rates of major bleeding and overall mortality. A post hoc analysis of CLOT trial [35] showed that treatment with dalteparin was associated with improved overall survival in patients without metastatic disease (probability of death at 12 months: 20 % in the dalteparin group vs. 36 % in the VKA arm, HR 0.50; 95 % CI 0.27–0.95; *p* = 0.03) compared to AVK treatment. In the largest one of these trials, the CLOT study, dalteparin was administered at a full dose for the first month followed by a 25 % reduction in the treatment dose for the remaining 5 months. This LMWH dose reduction approach can be considered especially in patients with previous history of bleeding or with a higher risk of bleeding. LMWH offers additional advantages compared to VKA in the long-term management of cancer-related VTE: limited drug interaction and a shorter half-life that facilitates temporary interruption for invasive procedures or thrombocytopenia.

**Table 5:** Randomized trials comparing LMWH with AVK for cancer-related VTE secondary prophylaxis

Clinical trial/year	Study drug	*N*	Observation period	Recurrent VTE (VKA vs. LMWH)	Major bleeding (VKA vs. LMWH)	Mortality (VKA vs. LMWH)
CLOT 2003	Dalteparin 25 % LMWH dose reduction after 1 month	672	6 months	17 vs. 9 %; *p* = 0.02	4 vs. 6 %; *p* = 0.27	41 vs. 39 %; *p* = 0.53
CANTHANOX 2002	Enoxaparin full dose once daily	146	3 months	21.1 vs. 10.5 %; *p* = 0.09	16 vs. 7 %; *p* = 0.09	22.7 vs. 11.3 %; *p* = 0.07
ONCENOX 2006	Enoxaparin 2 full-dose schemes (twice and once daily)^a^	122	6 months	10 vs. 6.9 vs. 6.3. %; *p* = NS	2.9 vs. 6.5 vs. 11.1 %; *p* = NS	32.4 vs. 22.6 vs. 41.7 %; *p* = NS
LITE 2006	Tinzaparin full dose	200	3 months	16 vs, 7 %; *p* = 0.044	7 vs. 7 %; *p* = NS	19 vs. 20 %; *p* = NS

^a^AVK vs. enoxaparin 1 mg/KG/12 h and AVK vs. enoxaparin 1.5 mg/kg/day

Unfortunately, trials addressed to test the risk/benefit of extended duration of anticoagulation beyond 6 months are not available. Individualized decisions to tailor therapy according to the characteristics and preferences of each patient are challenging in clinical practice. Specific tools aimed to ‘predict VTE recurrence’ have been developed in recent years which may provide further support to clinicians in the decision to maintain extended anticoagulation beyond 6 months. Four risk assessment models combining clinical and biomarker variables are currently available to predict VTE recurrence, of which only one was specifically designed to predict recurrence in cancer patients known as Ottawa score [36] (Table 6).

**Table 6:** Risk assessment model for cancer-associated recurrent VTE: the Ottawa score

Risk factor	Points
Female sex	+1
Lung cancer	+1
Breast cancer	−1
TNM stage I	−2
Prior VTE	+1

−3 to 0 points: low VTE recurrence probability (≤4.5 % VTE recurrent risk)

1 to 3 points: high VTE recurrence probability (>19 % VTE recurrent risk)

#### Recommendation

The preferred agent for long-term anticoagulant treatment of VTE in cancer patients is LMWH for at least 6 months (level of evidence: grade 1A). Extended duration of anticoagulation therapy after 6 months should be considered for high-risk patients such as those with active cancer and those receiving chemotherapy. Beyond 6 months, patients should be re-evaluated frequently to assess the risk–benefit ratio of continuing anticoagulant therapy (level of evidence: grade 2C).

## Special situations in the management of cancer-associated VTE

### Treatment of recurrent VTE during anticoagulation therapy

**Fig. 1:**
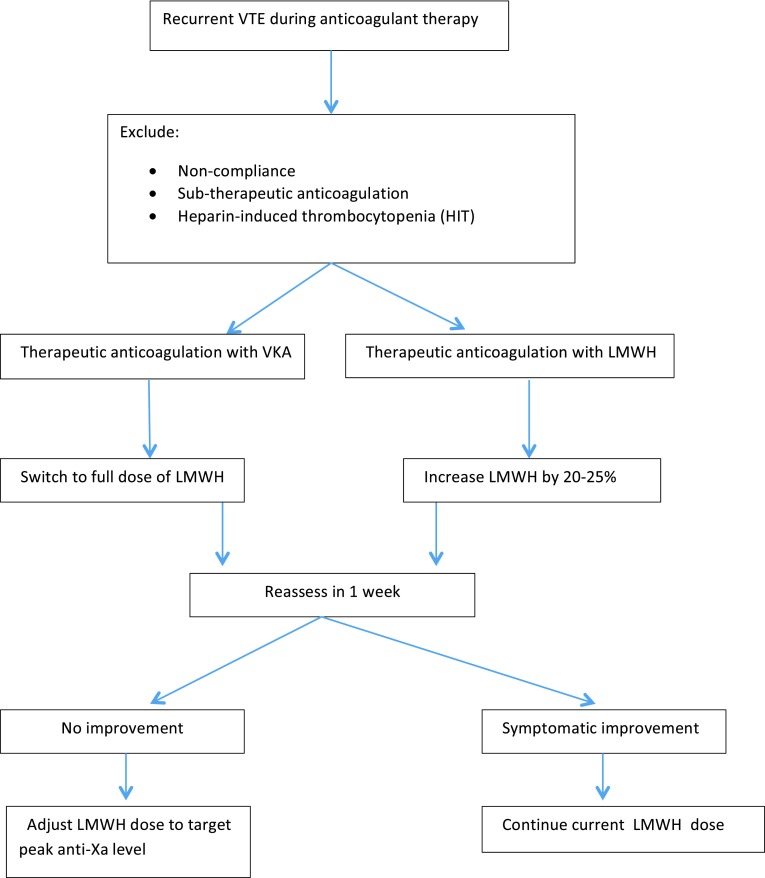
Management of recurrent cancer-associated thrombosis (CAT)

The reported rates of recurrent VTE during anticoagulant therapy in clinical trials are 7–10.5 % in patients treated with LMWH (Table 5). Gender, tumor type, TNM staging and prior history of VTE have been found to be risk factors for VTE recurrence in cancer patients. Cancer progression should also be considered in patients with recurrent VTE. Despite the high frequency and relevance of recurrent VTE in clinical practice, there is a lack of evidence to guide its management. An empirical approach for the management of recurrent VTE is proposed (Fig. 1). HIT in patients who were first exposed to LMWH or UFH, non-compliance and sub-therapeutic anticoagulant doses have to be ruled out and adjusted. If recurrent VTE occurs while receiving therapeutic anticoagulation VKA doses, it is recommended VKA be switched to LMWH. In patients who experience VTE recurrence despite therapeutic weight-adjusted LMWH, it is recommended to continue LMWH at a higher dose starting with an LMWH dose escalation by 20–25 %. If another VTE episode occurs after the first dose escalation, further dose increase or twice-daily dosing of LMWH is considered to be reasonable option. The use of peak anti-activated factor X (anti-factor Xa) levels may help to estimate further tailor LMWH escalation, although published evidence to support this strategy is lacking. The insertion of an inferior vena cava (IVC) filter in addition to anticoagulation is not currently recommended in preventing recurrent thrombosis. Data on the efficacy and safety of IVC filters in cancer patients are limited to retrospective single-center series and anecdotal reports. Available data from the PREPIC trial shows that IVC filter reduced the risk of PE but increased that of DVT and had no impact on patient survival [37]. Therefore, the use of IVC filters should be considered only in patients who cannot received anticoagulation or recurrent VTE occurs despite adequate anticoagulant therapy management, although evidence from prospective data are not available.

#### Recommendation

If recurrent VTE occurs with therapeutic weight-adjusted doses, a dose escalation could be performed (level of evidence: grade 2C).

IVC must be considered when anticoagulation is contraindicated or recurrent PE occurs despite adequate anticoagulant management (failure of anticoagulation). It is recommended to associate anticoagulation therapy to IVC, in those cases in which anticoagulation is contraindicated, it should be resumed as soon as possible (level of evidence: grade 2C).

### Thrombocytopenia

The optimal dosing of LMWH in the thrombocytopenic patient has not been properly investigated in clinical trials. The recommended approach by expert consensus is to maintain full-dose anticoagulation for platelet count >50 × 10^9^/L. For platelet counts between 20 and 50 × 10^9^/L, half-dose or prophylaxis dose of LMWH and close monitoring. And for platelet count <20 × 109/L, it is recommended to hold anticoagulation. In the acute period after VTE (first month), the risk of recurrent VTE is higher and platelet transfusion to maintain platelet counts >50 × 10^9^ should be considered to allow maintaining a full-dose anticoagulant treatment.

### Anticoagulation in special situations: obesity, renal impairment and elderly

LMWH must be used with caution in obese and elderly patients and in those with renal insufficiency. Due to their predominantly renal elimination, LMWH accumulation is expected with long-term use in those with creatinine clearance <30 mL/min. Obese patients and elderly patients have a lower proportion of lean body mass as a percentage of total body weight. As a result, LMWH dosing based on total body weight could cause supra-therapeutic anticoagulation. LMWH appropriate dosing in patients with severe renal impairment (CrCl < 30 mL/min) is uncertain and must be cautiously used with regular monitoring of renal function and anti-Xa levels with dosage adjustment. UFH and AVK therapy are not reliant on renal elimination and are considered to be an alternative approach in this setting for long-term treatment according to expert recommendations if anti-factor Xa is not available.

### Anticoagulation and central nervous system malignancies

VTE is common perioperatively and through the course of brain tumor therapy with an incidence of 20–30 % per year of survival. These patients also have the risk of potentially serious and life-threatening intracranial bleeding. No randomized controlled trials exist to evaluate the best management of VTE in patients with primary or metastatic intracranial tumors. Nonetheless, small retrospective studies indicate that anticoagulation can be safely used in this challenging setting and therefore standard anticoagulation is recommended in these patients.

### Management of splanchnic vein thrombosis

Splanchnic vein thrombosis (SVT), involving the portal, splenic, mesenteric, or hepatic veins is a common event in cancer patients. Treatment of SVT is a clinical challenge due to heterogeneity of clinical presentations, increased bleeding risk, and lack of evidence from clinical trials. In recent years, different advances in diagnostic imaging techniques have led to an increase in the diagnosis of incidental SVT. Patients with acute, symptomatic SVT with low risk of bleeding should be treated with anticoagulation therapy. For patients with asymptomatic incidentally detected SVT, there is no specific guidance on treatment. It is reasonable to withhold anticoagulation if the patient is truly asymptomatic, especially if radiologic evidence indicates that the thrombus is chronic. Closely monitoring imaging is recommended to detect thrombus progression if anticoagulation is not given.

#### Recommendation

Acute symptomatic SVT should be treated with anticoagulant therapy. Treatment of asymptomatic incidentally detected SVT should be individualized in every patient (level of evidence: grade 2C).

### Prevention of PTS

PTS is a frequent complication (20–50 %) within the first 2 years after DVT and is a common cause of morbidity. Clinical manifestations are usually leg pain, edema, swelling and eczematous skin changes which may range from mild complaints to intense pain intervening with daily activities. The only method that has been shown to be potentially effective in the prevention of PTS is the use of graduated compression stockings. Previous small and non-placebo trials suggest a benefit of elastic compression stockings (ECS) to prevent PTS with an overall reduction of 50 % in severe PTS. A recent study published in 2014 (SOX trial) [38] randomized patients to ECS used for 2 years or placebo after a first proximal DVT. The incidence of PTS was 14.2 % in the experimental arm vs. 12.7 % in the placebo group (HR 1.13, 95 % CI 0.73–1.76; *p* = 0.58). The findings of the SOX trial do not support routine wearing of ECS after DVT for the prevention of PTS.

#### Recommendation

ECS is not recommended routinely to prevent PTS (level of evidence: grade 1B).

### VTE: Antiangiogenic therapy/anti-epidermal growth factor receptor (EFGR) therapy

Venous or arterial thromboembolism and bleeding are adverse events linked to angiogenesis inhibitors. Combination treatment with bevacizumab and chemotherapy compared with chemotherapy alone was associated with a modest but significant twofold increased risk of arterial thromboembolic disease (ATD), but the impact on the risk of VTE remains controversial [39, 40]. Development of ATD with bevacizumab treatment was associated with a prior arterial thromboembolic event in patients older than 65 years. The prophylactic use of acetylsalicylic acid is controversial. The start of treatment with bevacizumab after an ATD must be individualized, but the minimum interval recommended is 6 months. If an ATD takes place during treatment with bevacizumab, its use must be stopped permanently because the safety of resuming bevacizumab has not been studied. In patients who develop VTE during bevacizumab treatment, the minimum safety interval recommended to resume bevacizumab is 2 weeks with stable dose of anticoagulant treatment. If there is a grade 4 VTE episode during treatment with bevacizumab, its use must be permanently stopped. Recommendations regarding aflibercept (an anti-VEGF recombinant fusion protein) are similar to those with bevacizumab.

VEGF tyrosine kinase inhibitors (TKI) are used in multiple cancers. In a meta-analysis evaluating the risk of ATD associated with sunitinib and sorafenib, a significant threefold increase was observed [41]. These results may be related to the higher time exposure to sunitinib and sorafenib in the trials as confounding factor.

A meta-analysis that explored the risk of VTE with anti-EGFR drugs (either monoclonal antibodies-MoAbs- or TKIs) showed that anti-EFGR therapy increased by 32 % the risk of VTEs, but not of ATEs. This thrombotic risk is increased with cetuximab and panitumumab but not with gefitinib and erlotinib. The differences in the results between MoAbs and oral TKIs are still unknown, but the association of cetuximab and panitumumab with other cytotoxic agents probably matters [42].

### Anticoagulation in the absence of VTE to improve survival in cancer patients

The mechanism by which anticoagulation might provide a survival benefit beyond the prevention of VTE is unknown. Several clinical trials have tested anticoagulant therapy in cancer patients without VTE with survival as primary end point with inconclusive results [43–46]. The survival benefit may be dependent on the tumor and the extent of disease, being larger with localized disease and small-cell lung cancer.

#### Recommendation

Currently anticoagulant therapy in cancer patients without VTE with the intention to improve survival cannot be recommended (level of evidence: grade 1B).

### New oral anticoagulants and VTE in cancer patients

There are three (NOACs) currently marketed in Spain, dabigatran etexilate (direct thrombin inhibitor), rivaroxaban and apixaban (factor Xa inhibitors). NOACs have been studied in large randomized clinical trials for acute VTE treatment and for VTE prophylaxis. In these trials, the number of patients with active cancer enrolled was small. Due to the paucity of data in the oncology subgroup, the results of these trials cannot be generalized to cancer patients, and additional information is needed about the efficacy and safety of NOACs in this population. There is an additional concern about the drug interactions between NOACs and either chemotherapy or biological treatments.

#### Recommendation

NOACS are not recommended for the treatment of VTE in cancer patients (level of evidence: grade 1B).

### Anti-Xa monitoring

The determination of anti-Xa in blood is the method of choice for monitoring the therapeutic range of LMWH. Anti-Xa monitoring is not routinely recommended in LMWH treatment. There is only one randomized trial that compares adjusted vs. fixed doses of LMWH in the treatment of DVT. Treatment efficacy and hemorrhagic complications did not differ between the two groups [47]. In special populations like obese, renally impaired, elderly, thrombocytopenic or patients with high risk of bleeding anti-Xa is currently recommended.

#### Recommendation

Anti-Xa monitoring is not routinely recommended in LMWH treatment (level of evidence: grade 1B).
